# Postpartum Vaginal Blood Loss following Two Different Methods of Cervical Ripening

**DOI:** 10.1155/2017/1678265

**Published:** 2017-12-19

**Authors:** Okon Asuquo Okon, John Egede Ekabua

**Affiliations:** Department of Obstetrics and Gynecology, Faculty of Medicine, University of Calabar, Calabar, Cross River State, Nigeria

## Abstract

Eighty women undergoing induction of labor at the University of Calabar Teaching Hospital were recruited and randomly allocated into two treatment groups (40 each), to receive either serial 50 *µ*g doses of misoprostol or intracervical Foley catheter. Vaginal blood loss was collected and measured using an under buttocks plastic collection bag and by perineal pad weighing up to 6 hours postpartum. There were no significant differences between the two groups with respect to sociodemographic and obstetric characteristics. Comparison of blood loss in vaginal deliveries between the two groups revealed that subjects in the misoprostol group had significantly higher blood loss than subjects in the Foley catheter group (488 ± 222 versus 326 ± 106, *p*<0.05). In both groups, there was strong and statistically significant positive correlation between postpartum blood loss and induction delivery interval (*r*=0.75, *p*<0.0001; *r*=0.77, *p*<0.0001). There were no significant differences in maternal outcomes. In view of this, further study is required to ascertain if lower doses of misoprostol for induction of labor may result in lesser blood loss. This trial is registered with ISRCTN14479515.

## 1. Introduction

Induction of labor is one of the most important interventions in obstetric practice, and the epidemiology of induction of labor has changed over the years, with increase in the frequency of term induction of labor for various indications [1]. Incidence of 3% was reported in Sokoto [2] and 5% in Benin [3]; up to 23% has been documented in developed countries [2] and is still on the increase [4]. Postterm pregnancy however remains the commonest reason for induction of labor, but the gestational age at delivery for postdate pregnancies has declined generally from 42 to 41 weeks, corresponding with data showing a decreased risk of stillbirth when induction is done at 41 weeks' gestation [1, 4, 5].

There are various methods of induction of labor available. To achieve a favorable Bishop score and increase the chance of a successful induction, mechanical methods have been used, such as the placement of extraamniotic cervical catheter. This acts on the decidua and fetal membranes, enhancing the release of endogenous prostaglandins to initiate cervical ripening and softening, in addition to mechanically dilating the cervix [6].

Vaginal dinoprostone is the current gold standard method for cervical ripening and induction of labor, although much interest has been generated by the use of misoprostol in induction of labor since 1987, and it has been found to be efficacious and safe [7].

Misoprostol, a pharmacological analogue (methyl-ester) of prostaglandin E_1_ (PGE_1_), is cheap, easily stored at room temperature, and rapidly absorbed after vaginal administration and has few systemic side effects. Usually, it is used when the Bishop score is found to be below a score of 6.

The reported side effects of misoprostol induction include uterine tachysystole and hyper stimulation, fetal distress, and uterine rupture. However, little has been said about increased postpartum bleeding [8].

This appears to be quite relevant in our population at University of Calabar Teaching Hospital, where anemia is a common problem [9], as any significant amount of blood loss beyond what the individual mother/patient may be able to tolerate may compromise her hemodynamic status resulting in additional therapy, with increased healthcare costs.

This study aims to determine whether there are any differences in maternal blood loss when misoprostol as opposed to Foley's catheter is used for cervical ripening in induction of labor.

## 2. Materials and Methods

This study was done at The University of Calabar Teaching Hospital (UCTH). All patients for this study were recruited from the women who were admitted to the antenatal ward and labor ward of the hospital for induction of labor. Ethical approval was obtained from the Health Research and Ethics Committee of UCTH, Calabar (reference number: UCTH/HREC/33/104).

It included women who were at a gestational age of 37 completed weeks up to 41 completed weeks plus 3 days. Patients of the institution requiring cervical ripening (i.e., Bishop score of <5 using the modified Bishop score criteria) and induction of labor were eligible for this study if they had a live singleton fetus with cephalic presentation at term, intact membranes with no evidence of labor, and no contraindications to a vaginal delivery and up to the third parity.

Women with a history of uterine scar, twins, breech presentation, fetal anomalies, antepartum hemorrhage, polyhydramnios, and presence of uterine fibroids in pregnancy and a known allergy to prostaglandin preparations were excluded; also, women with anemia (defined as a hemoglobin level of less than 10.5 g/dl or a hematocrit level of less than 31%), bleeding disorders, or pelvic abnormalities were excluded from the study.

The aim and purpose of the study was explained to the women, and appropriate counselling was offered. Thereafter, informed written consent was obtained.

The antenatal records of the women were reviewed in addition to obtaining a detailed history and filling out an evaluation form (proforma) to obtain any other relevant data not contained in the antenatal records. Information extracted from the antenatal records included record of HIV and hepatitis screening done by the patient. The data collected by the proforma included the following: the treatment group and number of the patient, marital status, level of education, occupation, age, gravidity and parity, gestational age, and indication for induction. Thorough physical examination was done. Detailed sonographic examination to document fetal presentation, estimated fetal weight, and biophysical profile was carried out in all women.

An initial pelvic examination to evaluate the Bishop score and pelvic capacity was carried out, and those with detectable abnormalities were excluded. Eligible patients were then assigned to treatment groups by opening an opaque, sealed envelope that contained the results of computer-generated random numbers, to receive either misoprostol (Cytotec, Searle Pharmaceuticals, High Wycombe, Bucks, UK) or extraamniotic Foley catheter.

For the misoprostol group, using the hospital protocol, a 50 *µ*g tablet was inserted into the posterior fornix of the vagina, and it was repeated 6 hourly until an adequate contraction pattern (3–5 contractions in ten minutes, each lasting between 40 and 60 seconds), sufficient cervical ripening (Bishop score greater than 7 or cervical dilatation of at least 4 cm), or spontaneous rupture of membranes was achieved, or a maximum of four doses had been given.

In the extraamniotic Foley catheter group, a size 18 Foley Catheter (Agary Pharmaceutical, China) was inserted through the cervix into the extraamniotic space under aseptic conditions, and the bulb was inflated with 30 cm^3^ of sterile physiological saline or sterile water. The catheter was taped under gentle traction to the inner aspect of the woman's thigh. This was left in situ until spontaneously expelled, but not exceeding 12 hours when the balloon was deflated and the catheter was removed. They then proceeded to have synchronous fore-water amniotomy and incremental intravenous oxytocin titration. The hospital protocol for oxytocin administration is gravity fed intravenous infusion using 0.9% normal saline at a concentration of 10 mU/ml (10 IU of oxytocin in 1 litre of fluid) for primigravida and multigravida. It is commenced at a rate of 10 drops per minute (6.67 mU/min) and titrated by increasing the rate by 10 drops every 30 minutes, until adequate contractions are established (i.e., 3–5 contractions in ten minutes, each lasting between 40 and 60 seconds) or till a maximum rate of 60 drops per minute (40 mU/min) is reached.

In either group, induction of labor was considered to have failed, if no vaginal delivery was achieved after 12 hours from the commencement of the active phase of labor.

With the delivery of the baby, 10 international units of Syntocinon was given intramuscularly, and the placenta was delivered by controlled cord traction as the uterus contracted. The lower genital tract was then inspected for any lacerations, which were then sutured immediately. If an episiotomy was given, it was sutured immediately.

The procedure adopted for the assessment of blood loss in this study was a combination of two methods to allow for reasonable accuracy and reduction of bias to a minimum level. It was noted that the use of plastic bags for the collection of blood and hind waters introduced some level of inaccuracy into the measurement of the blood loss, as the volume to be measured also included some liquor. This however was expected for both arms, as the normal evaluation of blood lost after normal vaginal deliveries also carried this inherent error. The more accurate means of measurement was the packed cell volume. For the study, both methods were compared to arrive at a more logical conclusion, with respect to satisfactory methods for measurement of blood loss postpartum.

Once the fetal head had crowned, a plastic bag was placed under the patient to collect the blood and hind water following the delivery of the fetus and the placenta. It did not require sterilization and was used in the dorsal, lateral, or lithotomy positions. The bag was left in situ until the birth attendant was no longer concerned about blood loss, such as when a sanitary towel was applied to the vulva. Thereafter, the collected blood was poured into a graduated measuring cup, promptly read and recorded. The sanitary towel was left in place to collect blood lost per vagina until 6 hours postpartum and then weighed to determine the amount of blood lost. For women undergoing caesarean section, the use of suction tubes and bottles and weighing of the abdominal mops, gauze, and swabs were used to determine the amount of blood lost.

Ideally double blinding and use of placebos are the standard for any randomized controlled trial; however, by the nature of the study interventions, it was not possible to blind the researcher and subjects. So, an open labelling of the participants after adequate randomization was adopted.

There were many factors that could have affected the internal validity of this study. These factors were included in the exclusion criteria.

Data were analyzed using CDC Epi Info^TM^-7.0.8.3 and SPSS-21^TM^; comparison was made using χ^2^ test, Student's *t*-test, and Fisher's exact test as appropriate. Differences were considered significant if *p*<0.05.

## 3. Results

During the study period, there were 1,674 deliveries in the hospital; of these, 218 were admitted for and had induction of labor, thus giving an induction of labor rate of 13%. Data were obtained from 80 subjects, consisting of 40 subjects in each of two groups (1 and 2) that were exposed to misoprostol and Foley's Catheter, respectively. Mean age of subjects was 30.0 ± 7.2 years, ranging from 16 to 47 years, with 20–29 years being the commonest age group (Table 1). There was no significant difference in the mean ages of the two groups (29.2 ± 5.6 versus 30.9 ± 8.5; *p*=0.31).

There was no significant difference in mean parity, mean gestational ages at induction of labor, and mean preinduction Bishop scores between the two groups (Table 2).

The mean number of doses of Cytotec used for subjects in group 1 was 1.2 ± 0.4 with a range of 1 to 3 doses, and a 10% failed induction rate. For subjects in group 2, the mean interval between insertion of Foley's catheter and when it fell off was 9.4 ± 2.6 hours, with a range of 3.25 to 12.0 hours. There was no subject whose catheter had to be removed after 12 hours; all had a favorable Bishop score and all parturient had artificial rupture of membrane done; only two subjects (5%) in group 2 had to have oxytocin augmentation, increased incrementally, up to a strength of 20 mU/min at the time of delivery; and none had a failed induction.

### 3.1. Comparison of Obstetric Outcome between Study Groups

In this study, there was no significant difference in the occurrence of vaginal and Caesarean deliveries between the two groups. Among subjects that had vaginal delivery, there was no statistically significant difference in the rates of instrumental delivery or in the rates of episiotomies (Table 3). Indications for caesarean section were fetal distress (3; 75%), and cephalopelvic disproportion (1; 25%), and indications for operative vaginal deliveries were maternal exhaustion, delayed second stage, suspicion of fetal compromise, and imminent perineal laceration.

### 3.2. Blood Loss following Induction of Labor

The mean blood loss following vaginal delivery was 318.0 ± 131.7 ml, ranging from 200 to 1,200 ml.

Comparison based on vaginal deliveries between the two groups reveals that the mean blood loss for those in group 1 was 488 ± 222 and for group 2 was 326 ± 106, *p*=0.002 (Table 4).

Further analysis based on spontaneous vertex vaginal deliveries showed that there was significant difference between the groups (480 ± 275 versus 305 ± 96.7, *p*<0.0001); however, for deliveries associated with episiotomy and/or instrumental delivery, there was no significant difference in mean blood loss between the groups (497 ± 151.5 versus 382 ± 115.7, *p*>0.58) (Table 5).

### 3.3. Correlation between Postpartum Blood Loss and Relevant Predictors

**Figure 1:**
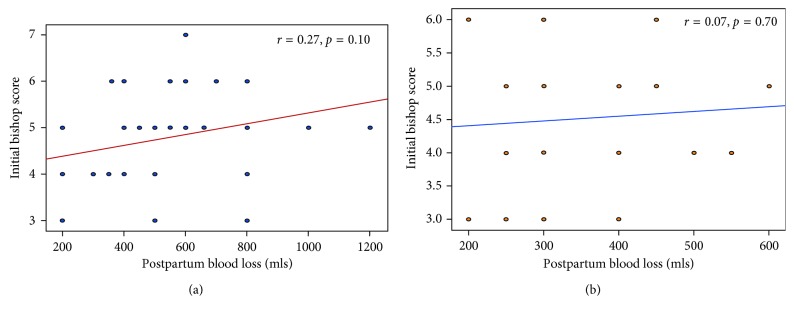
(a) Correlation between postpartum blood loss and the initial Bishop score in group 1 (*N* = 40). (b) Correlation between postpartum blood loss and the initial Bishop score in group 2 (*N* = 40).

**Figure 2:**
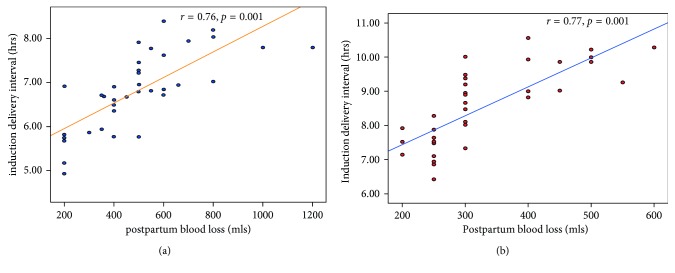
(a) Correlation between postpartum blood loss and induction delivery interval in group 1 (*N* = 40). (b) Correlation between postpartum blood loss and induction delivery interval in group 2 (*N* = 40).

Both groups showed strong and statistically significant positive correlation between postpartum blood loss and induction delivery interval (*r*=0.75, *p*<0.0001; *r*=0.77, *p*<0.0001). However, for both groups, there was no significant correlation between postpartum blood loss and preinduction Bishop scores (Table 6, Figures 1–2).

The overall mean birth weight was 3238.75 g ± 455 g, with a range of 2200 g to 4500 g. There was no significant difference in the birth weights of the babies between the two groups (3227.5 ± 433 versus 3250 ± 482.5, *p*>0.99) (Table 7).

## 4. Discussion

The findings of this study revealed that the use of misoprostol compared to Foley catheter for induction of labor is associated with increased blood loss postpartum, following vaginal delivery.

The groups had similar demographic characteristics. There was no significant difference in the indications for induction of labor and initial cervical Bishop score prior to induction of labor. These findings were in conformity with those of Adeniji et al. [10] who also found no differences between the study groups in their report with respect to their demographic characteristics and indications for induction of labor.

Although the preinduction Bishop scores were similar for both groups, misoprostol was associated with a shorter induction-contraction time and a shorter induction-delivery interval. This observed difference may be due to the higher dosage of the medication used, in other words making more of the medicine available at the site of primary effect and improving its profile of activity. It may also be possibly related to its being an analogue prostaglandin, which confers a higher potency [11].

The induction delivery interval of 6.89 ± 0.89 versus 8.63 ± 1.18 hours was significantly greater for subjects in group 2 in this study and was similar to the findings of Owolabi in Ile-Ife [12], Nigeria, who reported similarly significantly increased induction delivery interval in the Foley catheter group.

In this study, it was found that the blood loss after misoprostol induction was significantly higher than that after Foley catheter (*p*<0.05) with respect to vaginal delivery. For both groups, there was significant positive correlation between the induction delivery interval and blood loss at delivery. However, there was no significant correlation found between postpartum blood loss and the initial Bishop score.

Cervical ripening is the only discernible indicator that labor has drawn near and that induction may be successful [13, 14], although the exact mechanism leading to physiological cervical ripening and labor is not known.

It is plausible that the rapid progress through labor may possibly obviate the adequate expression of other regulatory molecules that are required for effective control of blood loss after placental delivery [8]. Additionally, possible excessive collagenolysis [11] near the cervix and lower uterine segment induced by misoprostol induction may result in undue fragility of tissues in this area and may interfere with effective retraction required for arrest of bleeding after labor.

The 180 ml mean difference in blood loss between the two groups is significant, and it shows that misoprostol is associated with more blood loss following its use for cervical ripening and induction of labor.

Most of the babies delivered by the parturient in this study were average weight babies with birth weight ranging mostly between 2500 g and 3900 g, suggesting that macrosomia was not a major factor for the observed bleeding differences.

The management of the third stage of labor was standardized for all parturient; therefore, no difference in management could explain the difference in blood loss observed.

## 5. Conclusion

In this study, misoprostol resulted in onset of contractions and in delivery much more quickly than with Foley's catheter. Comparison of blood loss at vaginal delivery between the two groups revealed that misoprostol resulted in greater blood loss than Foley catheter.

Intravaginal misoprostol when compared with Foley's catheter is a more effective alternative for induction of labor. Misoprostol use was associated with higher blood loss in this study. However, lower doses of misoprostol may be more appropriate, instead of 50 mcg, as used in this hospital for patients who may have chronic anemia (such as sickle cell disease patients), in whom even minor blood loss may significantly impact on their hemodynamic system [15].

## 6. Limitations

Blinding of subjects could not be effected due to the study design. This can create bias and mask cause and effect relationships or suggest correlations where there are none.

Though extra precaution was taken to exclude all external causal effect, the use of collector bags for blood and hind water collection and the measurement of both introduced the possibility of an error in the measured blood lost postpartum for all the women in both arms of the study.

## Figures and Tables

**Table 1:** Comparison of age characteristics of subjects (*N* = 80).

Variable	Group 1, *n* (%)	Group 2, *n* (%)	Chi-square test	*p* value
Age groups (in years)				
<20	2 (5)	3 (7.5)	Fisher's exact test	0.11
20–29	20 (50)	18 (45)		
30–39	17 (42.5)	11 (27.5)		
40–49	1 (2.5)	8 (20.0)		
Total	40 (100)	40 (100)		

**Table 2:** Comparison of obstetric characteristics between the groups (*N* = 80).

Variable	Group 1, mean (SD)	Group 2, mean (SD)	*t*-test	*p* value
Parity	1.63 (1.66)	1.58 (1.43)	1.44	0.89
Gestational age (in weeks)	39.7 (1.37)	40.1 (1.36)	1.50	0.14
Initial Bishop score	4.73 (0.96)	4.48 (1.1)	1.10	0.28

**Table 3:** Comparison of obstetric outcome between study groups (*N* = 80).

Variable	Group 1, *n* (%)	Group 2, *n* (%)	Test statistic	*p* value
*Mode of delivery*				
Vaginal	36 (90)	40 (100)	—	0.06^∗^
Caesarean	4 (10)	0 (0)		
*Type of vaginal delivery*				
Normal	30 (83.3)	34 (85)	*X* ^2^ = 0.01	0.91
Instrumental	6 (16.7)	6 (15)		
*Episiotomy during vaginal delivery*				
Yes	15 (41.7)	10 (25)	*X* ^2^ = 1.7	0.2
No	21 (58.3)	30 (75)		

^∗^Fisher's exact test.

**Table 4:** Blood loss analysis by vaginal delivery.

Variable	Group 1 (*n* = 36)	Group 2 (*n* = 40)	Test	*p* value
Vaginal delivery, mean ± SD	488 ± 222	326 ± 106	9.85	0.002

**Table 5:** Blood loss analysis by type of vaginal delivery.

Variable	Group 1	Group 2	Test	*p* value
Normal Vaginal delivery	*n* = 19	*n* = 29	16.2	<0.0001
Mean ± SD	480 ± 275	305 ± 96.7		
Vaginal delivery with episiotomy or instrument	*n* = 17	*n* = 11	0.307	0.584
Mean ± SD	497 ± 151.5	382 ± 115.7		

**Table 6:** Correlation between postpartum blood loss and Bishop scores, and induction delivery interval in the two study groups (*N* = 76).

Postpartum blood loss	Group 1		Group 2	
	Pearson *r*	*p* value	Pearson *r*	*p* value
Initial Bishop score	0.28	0.11	0.07	0.70
Induction delivery interval (hrs)	0.75	<0.0001	0.77	<0.0001

**Table 7:** Birth weight analysis.

	Group 1 (*n* = 40)	Group 2 (*n* = 40)	Test	*p* value
Mean ± SD	3227.5 ± 433	3250 ± 482.5	0.001	0.992
Range	2500–4300	2200–4500	—	—
LBW	0	1	—	0.99
Fetal macrosomia	1	2	—	0.97^∗^
Overall	3238.75 ± 455, range 2200–4500			

^∗^Fisher's exact test.
